# Too many cooks in the kitchen: HPV driven carcinogenesis – The result of collaboration or competition?

**DOI:** 10.1016/j.tvr.2024.200311

**Published:** 2024-12-27

**Authors:** Weimer Kathleen

**Affiliations:** IGBMC – CBI: Institut de génétique et de biologie moléculaire et cellulaire, Centre de biologie intégrative, 1 rue Laurent Fries, Illkirch-Graffenstaden, BP 10142, 67404, France

**Keywords:** HPV, E6, Oncoproteins, Carcinogenesis, Papillomavirus, Oncogenesis

## Abstract

Infection by Human Papillomaviruses accounts for the most widespread sexually transmitted infection worldwide. Clinical presentation of these infections can range from subclinical and asymptomatic to anogenital cancers, with the latter associated with persistent infection over a significant period of time. Of the over 200 isotypes of the human virus identified, a subset of these has been characterized as high-risk due to their ability to induce oncogenesis. At the core of Papillomavirus pathogenesis sits three virally encoded oncoproteins: E5, E6, and E7. In this review we will discuss the respective roles of these proteins and how they contribute to carcinogenesis, evaluating key distinguishing features that separate them from their low-risk counterparts. Furthermore, we will consider the complex relationship between this trio and how their interwoven functional networks underpin the development of cancer.

## 1 An introduction to HPV life cycle and pathologies

**Fig. 1:**
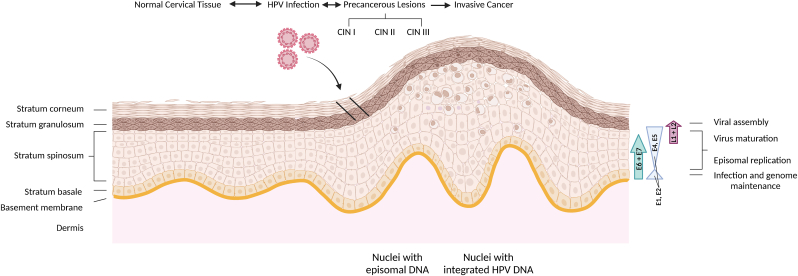
The progression of HPV induced cancer in epithelial tissue. Schematic representation of epithelial tissue reorganization in the development of HPV related cancers. On the left epithelial layers are defined. HPV enters through microabrasions or the junction between the endo- and ecto-cervix establishing low copy number episomal replication in basal cells. As infected cells climb through the stratum of the epithelial tissue, changes in viral protein levels can be viewed on the right side with their corresponding stages of the viral life cycle. Following infection precancerous lesions, graded CINI-III, may progress to invasive cancer as the result of persistent infection over the course of up to 20 years. This transition is marked by a decrease in episomal DNA and an increase of cells containing integrated viral DNA. Created in BioRender. Weimer, K. (2024) https://BioRender.com/z51i407.

Research efforts to understand Human Papillomaviruses (HPVs) often focus on high-risk HPVs (hr-HPVs), particularly the prototypical HPV16, due to their role in cervical and other cancers [1,2]. These investigations have uncovered a universal pattern of viral gene expression that can be extended, with some modification, across different HPV groups [3]. Infection commences when the virus enters cells of the basal layer, presumably gaining access to the epithelium through microabrasions [4] or, in the case of cervical infection, by traversing the squamocolumnar junction between the endo- and ectocervix [5]. Upon access, an effective infection is established in dividing basal epithelial cells (Fig. 1) [6,7]. This first stage involves viral DNA replication and genome maintenance [8]. During this step, the viral genome, in complex with L1 and L2, is trafficked to the nucleus [9,10] where early replication is initiated, producing between 50 and 100 episomal copies per nucleus [11]. In parallel, a period of cell proliferation is provoked, creating a layer of basal cells harboring replicated episomes [4]. Viral protein expression remains low during this phase due to E2-mediated repression of the early promoter in efforts to subvert immune detection [12]. This makes E1 and E2 the primary players, as they sequester host machinery for DNA replication [13,14]. Following ongoing division of infected cells, some progeny remain in the basal layer, acting as an episomal reserve, while others ascend, migrating through the epithelial strata, towards the productive phase of the viral life cycle [15]. It is here, in the suprabasal layer, that viral oncoproteins E5, E6, and E7 assume more prominent roles, postponing terminal differentiation and blocking departure from the cell cycle, thereby stabilizing an environment that promotes genome amplification at high copy number [[16], [17], [18]]. At the summit of the epithelium, after terminal differentiation and the expression of late genes L1, L2, and E1^4, the HPV life cycle concludes with virion assembly in the nucleus followed by subsequent release from the epithelial surface [19].

Often HPV infections are asymptomatic, cleared by the host immune system within a year or two devoid of any indication of infection [20]. In some cases, persistent infection may occur, and if these infections are not readily resolved by the immune system, they may result in cancer progression (Fig. 1) [21]. In this progression, cervical cancer is preceded by cervical disease, or precancerous lesions, a common occurrence that is the consequence of HPV infection [8,22]. These precancerous lesions, or “cervical intraepithelial neoplasia” (CIN), are scored into three grades: CINI, CINII, and CINIII [23,24]. In the CINI stage, HPV infection is still considered transient and it is not uncommon to detect infections by multiple HPV types [25]. The prognosis for cervical cancer development from CINI is low, with HPV infections often resolving. However, progression from CINI to CINIII is considered indicative of underlying cancer formation [26]. Of HPV-infected women, approximately 10 % show signs of one of these stages, representing oncogenic transformation of the cervix [27]. However, it is important to note that hr-HPV induced oncogenesis is an uncommon occurrence [2,8]. Oncogenic transformation is predominantly confined to the site of the squamocolumnar junction within a small region of metaplastic squamous epithelium, deemed the “transformation zone”, where cells appear to be especially susceptible to hr-HPVs [4,5,28,29]. Further still, carcinogenesis is not an ideal outcome for the virus as transformed cells are differentially defective, ultimately terminating viral replication, which is tightly tethered to this process [6,30,31]. With this in mind remission from CIN states is frequent, occurring spontaneously in approximately 80–90 % of CINI cases and up to 60 % of CINIII cases [26].

In the event that tumorigenesis does occur, it is instigated by the increased expression of virally encoded oncoproteins, principally E6 and E7. Activities of these proteins involve targeting host factors to induce cell growth, chromosomal instability, and decreased differentiation. Upregulation of these proteins occurs in the wake of HPV genome integration into that of the host [32]. Many molecular models explaining integration have been proposed, but it remains a complex process, further compounded by host co-factors, making it difficult to reach a clear consensus [27]. Several “hot spots” within the host genome have been suggested as preferential sites for HPV integration [[33], [34], [35]], but ambiguity remains regarding when integration is initiated and its contribution to carcinogenesis, with evidence of integration found in all grades of CIN [[36], [37], [38], [39]]. However, it is understood that integration events diverge from the normal HPV replication cycle, leading to an abortive infection where the production of virions arrests despite ongoing synthesis of viral proteins [15,40]. In the process of integration, expression of E2 is lost, alleviating repression of E6 and E7 [8,27]. This has catastrophic consequences for the cell, as the aberrant activity of these proteins exceeds their attempts to evade immune detection and sustain the viral lifecycle, instead driving immortalization and malignancy.

## 2 HPV oncoproteins – E5, E6, and E7

At the heart of HPV pathogenesis is a triad of oncoproteins: E5, E6, and E7 [41]. While these proteins are known for onsetting oncogenesis, their primary roles are necessary for the HPV life cycle [8]. These proteins play critical roles in regulating the host immune response, circumventing detection by disrupting gene expression, hijacking cellular networks via protein-protein interactions, inducing posttranslational modifications, and facilitating the cellular trafficking of key host immune modulators [42,43]. Studies monitoring E6 and E7 mRNA in middle and lower layers of the epithelium exhibited elevated levels [[44], [45], [46]] compared to biomarker studies measuring oncoprotein expression in the upper stratum which demonstrated diminished levels [47]. Taken together, considering the early roles of these proteins, such as the requirement of E6 for episomal genome maintenance [16,48,49] and E7 activation of the G1/S checkpoint supporting viral replication [50], it indicates that the activity of these proteins is likely most important during early infection. Despite this, their dysregulation is viewed as deterministic of cancer progression as their efforts to drive the viral life cycle coincidently prompts a persistent pro-proliferative cellular state [51]. While continuous oncoprotein expression is necessary to uphold the transformed phenotype, it alone is not sufficient for transformation, and given that the majority of infected cells do not progress to cancer, this reaffirms that other factors must influence carcinogenesis [52]. Upregulation of oncoprotein expression appears to follow integration and can confer a selective growth advantage to cells [53,54]. However, as it is not the predominant phenotype, this appears to be a fate stimulated by interactions with the host as opposed to the virus itself. Here we will review each oncoprotein, evaluating key interactions with host factors, with an emphasis on E6, to highlight how this interplay can lead to the establishment of cancer.

### 2.1 E5—

The three early genes—E5, E6, and E7 have been classified as oncoproteins due to their functions in driving carcinogenesis [55]. However, not all HPVs encode E5 with types from the Beta, Gamma, and Mu genera lacking the E5 ORF entirely [56]. As such, primary transformative and oncogenic functions have largely been attributed to E6 and E7 with E5 reduced to a supporting role. However, after demonstrating the protein's ability to induce anchorage-independent growth in murine fibroblasts and keratinocytes [[57], [58], [59]] E5 earned its class as a *bona fide* oncoprotein.

More recently, evolutionary studies of genital HPVs have given rise to the hypothesis that carcinogenic HPVs all stem from a shared lineage and encode E5 [[60], [61], [62]]. Further classification of the E5 ORF into four groups—alpha, beta, gamma, and delta—revealed a clustering based on clinical manifestations and identified a subset of 10 E5 encoding low-risk (lr-HPVs) which elicit benign venereal warts [63]. Of these, E5β HPVs are the only group associated with cutaneous lesions. Additionally, analysis of differentially active 16E5 variants in vitro drew an association between variants displaying the greatest mitogenic activity and those most commonly detected in the population and in cervical lesions [64]. Taken together, this suggests that while E5 is not essential for the viral life cycle, it does confer certain advantages that favor infection and transformation [65]. Even so, the exact functions of E5 remain elusive, and its contributions have been limited to the early stages of tumorigenesis [51,[65], [66], [67]], leaving the protein primarily credited with augmenting the transformative effects of E7 and working in conjunction with E6 and E7 to drive malignancy [[68], [69], [70], [71], [72], [73]]. Efforts to determine the role of E5 have been complicated by the challenging biochemical nature of the protein, making it difficult to produce and study in the lab, along with a lack of specific antibodies that impedes the study of HPV-infected cells expressing endogenous levels of E5.

The HPV16 E5 protein is the most well-characterized of the E5 proteins, forming an 83 amino-acid polypeptide [74] with other E5 proteins varying in size from 40 to 85 amino acids [75]. These proteins are abundant in hydrophobic amino acids gathered into a multi-pass transmembrane protein with a cytosolic C-terminus [[76], [77], [78]]. Given these characteristics, it has been suggested that E5 belongs to a family of proteins, known as viroporins [79]— a group of viral membrane proteins characterized by the presence of at least one amphipathic helix as part of a hydrophobic domain that undergoes membrane insertion [80]. As viroporins homo-oligomerize, the amphipathic helices arrange themselves as a membrane-spanning hydrophilic central pore with hydrophobic residues facing outwards, interacting with the lipid bilayer [81]. This assembly acts as a channel, facilitating the passage of small molecules and ions thus allowing them to regulate ion homeostasis. While further study is needed to determine the ion selectivity of the E5 channel [82], it has been demonstrated that E5 oligomerizes in cells and forms a functional pore structure in liposomes [80]. Comparative analysis of lr- and hr-HPV E5 sequences denotes a difference in the conservation of two amino acids located at the end of the sequence [83]. This region corresponds to a predicted transmembrane helix where hr-HPVs retain histidine and alanine in place of the tyrosine and isoleucine found in lr-HPVs. While small hydrophobic residues have high packing values and are common within transmembrane helices, charged residues are less frequent [84]. An investigation of other viroporins evaluated the structural impact of a HxxxW motif. While terminal tryptophans are often described as anchoring residues for integral membrane structures [85], this study established that the H residue creates a change in tilt angle of the helix [86]. This change in tilt not only accommodates the larger size of the residue, but also increases solvent accessibility which may stimulate changes in ion selectivity [87]. Furthermore, since this tilt shift occurs even in the absence of a tryptophan it is likely histidine has a similar impact on hr-HPV E5s. Finally meta-analysis of trends in the amino acid composition of transmembrane helices suggests that increased sequence complexity is correlated with functionality. As such, transmembrane helices primarily composed of simple hydrophobic amino acids will mainly act as anchors compared to those with more diversified sequences which are associated with functionality [88]. Taken together, this suggests structural and functional differences in the E5 proteins of high and low risk types, however, further study is necessary to solidify this theory.

Furthermore, overexpression studies show that E5 localizes primarily to the Endoplasmic Reticulum (ER) and Golgi Apparatus (GA) [89,90], potentially associating with the plasma membrane as well, hinting towards a role in the trafficking of cytoplasmic membrane proteins [67,91]. In addition, a myriad of other functions has been described for E5 [63], including but not limited to, stimulation of the epidermal growth factor receptor (EGFR) signaling cascade [59,92], altering apoptotic response [93], inducing changes in membrane lipid composition [94,95], and blocking trafficking of major histocompatibility complexes (MHC) classes -I and -II [91,96]. Due to our limited understanding of E5 function, it is difficult to pinpoint exactly where it intersects with its counterparts, E6 and E7. That said, the evidence that E5 augments effects of E6 and E7 likely indicates a regulatory network underpinning their functionality.

### 2.2 E6—

The discovery that, in cervical tumors and cervical cancer-derived cell lines, the E6 ORF was retained and expressed even years after initial transformation led to speculation about its role as an oncoprotein [[97], [98], [99]]. Its intrinsic ability to transform cells was verified in several models [[100], [101], [102], [103], [104], [105]], however not all E6 proteins possess these capacities. While E6s of hr-HPVs have proven sufficient for transformation, lr-HPVs are not capable of transforming primary cells [106]. Additionally, in the case of hr-HPVs, E6 is considered to have weakly transformative capabilities, working in tandem with E7 to establish carcinogenesis. This has given rise to the paradigm that E7 operates as the founder of tumorigenesis while E6 perpetuates malignancy [107]. Supporting this theory is the evidence that immortalization of primary human keratinocytes requires the full viral genome intact, containing both E6 and E7 ORFs [108].

E6 proteins are small proteins approximately 150 amino acids in length [109], and fold into two domains, E6N and E6C, named according to their distal location at the N- or C-terminus respectively. Each domain assumes a structure consisting of a triple-stranded β-sheet and two short helices with another short helix bridging the two regions [110,111]. These proteins possess a total of four CXXC zinc binding motifs arranged into two pairs, forming two zinc finger domains that are essential for E6 activity [[112], [113], [114], [115]]. At its core, E6 forms a charged, hydrophobic binding groove aptly termed the “LxxLL binding pocket” due to its ability to recognize and bind a short linear LxxLL motif, normally found in the disordered regions of proteins within acidic peptides that arrange as a helix upon contact [[116], [117], [118], [119], [120], [121]]. Both lr- and hr-HPVs adopt this core fold, allowing them to attract LxxLL partners, but variations in binding profiles have been described, leading to their subcategorization. This dichotomy divides E6 proteins that utilize the LxxLL pocket to associate with the cellular E3 ubiquitin ligase, E6-Associated Protein (E6AP), a characteristic shared by Alpha, DyoDelta, Dyopi, Omega, and Omikron Papillomaviruses, from ones that interact with the Notch co-activator Mastermind Like 1 (MAML-1),as seen in the remaining genera [[122], [123], [124], [125], [126]]. This disrupts the traditional convention of using tropism to cluster HPV types as Alpha and Beta genera contain types responsible for both cutaneous and mucosal infections. Sequence and structural analysis of E6 contact residues within the LxxLL pocket revealed a lack of conservation, suggesting some E6 proteins may target multiple LxxLL partners, but it does not clarify the selectivity between E6AP and MAML-1. Additionally, functional assays demonstrated that E6 binding to E6AP alone is not sufficient to induce its degradation [122,127]. As such, there are low-risk α-HPVs which associate with E6AP but do not degrade it. Conversely, some β-HPVs have been linked to nonmelanoma skin cancers as E6 represses Notch trans-activation through its interaction with MAML-1 [126]. Given that the Notch signaling pathway is a critical determinant in keratinocyte differentiation and cell cycle arrest, it represents a key target for the HPV life cycle. Taken together, this demonstrates the importance of the nature of E6 interactions with their host factors in addition to the targets themselves. Finally, at the C-terminal of hr-HPVs a PDZ (PSD-95/DLG/ZO-1) binding motif (PBM) can be found [128]. This motif is considered a class I PBM formed by a X-S/T-X-V/L consensus sequence [129] which recognizes and binds PDZ containing partners [130]. Many PDZ proteins contain multiple copies of the domain in addition to other protein-protein interaction motifs, allowing them to serve as interaction hubs, erecting scaffolding for the assembly of multi-protein complexes involved in an array of cellular functions [131]. This structural distinction between lr-HPVs and hr-HPVs also distinguishes the group functionally. When trying to understand differences between viruses of the two groups, genome analysis has demonstrated a high degree of conservation between virus types. This makes the PBM of hr-HPVs a defining feature which can be used as a molecular signature to indicate oncogenic potential [[132], [133], [134]].

**Fig. 2:**
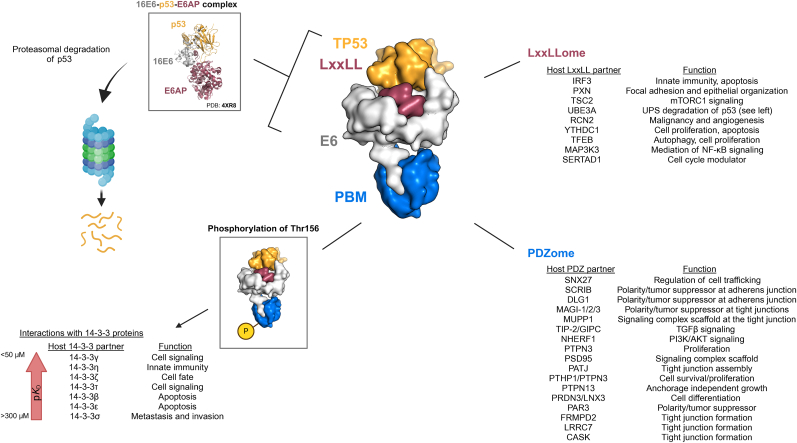
Interactions of hr-HPV E6. The canonical hr-HPV E6 structure is shown with interfaces labelled as follows: LxxLL binding pocket is highlighted in raspberry, the TP53 (p53) binding interface is highlighted in gold, the C-terminal PBM is highlighted in blue, and the remaining E6 surface is shown in grey. In the upper left-hand box, the structure of the tertiary 16E6-p53-E6AP complex is displayed (PDB: 4XR8). The proteasomal degradation of p53 is mediated by this complex. In the lower left-hand box, a diagram of E6 modified by phosphorylation of T156 is shown. This introduces interactions with members of the 14-3-3 family with their potential functions in cancer listed. An affinity scale is represented by a red arrow, indicating the strength of interactions which range from 300 μM–50 μM based on fragmentomic in vitro binding assays although only E6 binding to 14-3-3ζ has been functionally implicated *in vivo*. To the right interactions with LxxLL partners (top right) and PDZ partners (bottom right) are listed and referred to the LxxLLome and PDZome respectively. Created in BioRender. Weimer, K. (2024) https://BioRender.com/m12c626.

Utilizing its various protein interfaces E6 is able to target an array of host factors involved in diverse cellular processes (Fig. 2) and disrupt cellular function despite total lack of any inherent enzymatic activity [15]. The profound effect of E6 on cellular function is owed, in part, to its association with E6AP, which can lead to the recruitment and degradation of targets such as p53 [135,136] and some PDZ proteins [[137], [138], [139]]. Meanwhile, other studies have implicated the involvement of a ubiquitin ligase other than E6AP in the degradation of some PDZs [140,141]. Successful sequestration of p53 occurs after E6 undergoes conformational changes induced by E6AP binding, thus revealing an additional interface for direct p53 interaction [[142], [143], [144]]. Based on this model of tertiary complex formation, brought through binding-induced conformational changes, and our own E6 binding studies (data not yet published), E6 may interact through other intermediaries in a similar manner, leading to other unexplored interfaces. This mechanism could also underpin the promiscuous binding profiles exhibited by many E6 proteins as well as their diversity despite structural conservation. To this end, many interatomic studies [[145], [146], [147], [148], [149], [150], [151], [152], [153], [154]] have sought to characterize E6 interactions with aims of understanding their contributions to cancer development [155]. Comparison of E6 interactions from lr- and hr-HPVs revealed overlap in cellular targets, indicating some advantage in supporting the viral life cycle [156]. This overlap is also shared by oncoproteins of the same HPV type, suggesting that they are critical targets and that the accumulation of cellular effects from multiple successful viral attacks likely contributes to cancer development [157,158].

### 2.3 Other forms of E6 –

#### 2.3.1 Phospho-E6

We've discussed that the E6 C-terminal PBM acts as a critical determinant in distinguishing hr- and lr-HPVs, however, this region is not restricted to its interactions with PDZ partners, displaying dual functionality as a phospho-protein. Within the PBM, situated at T156, is a phospho-acceptor site that, upon modification, transitions E6 PBM targeting from interactions with PDZs in favor of associations with members of the 14-3-3 protein family [159,160]. Early studies asserted E6 phosphorylation was facilitated by either protein kinase A (PKA) or AKT, recognizing the phospho-site in a sequence dependent manner [161,162]. Further exploration revealed a correlation between DNA damage and increased levels of phospho-E6, linking the protein to kinases involved in regulating the cellular stress response, such as DNA PK [163].

Much remains to be understood regarding the role of the 14-3-3 protein family in HPV related pathogenesis. There are seven human isoforms of 14-3-3 proteins and to date, phospho-E6 has been shown to interact directly with 14-3-3ζ which stabilizes levels of E6 [161,164,165]. In general, this family is implicated in nearly every facet of cellular function and they are particularly known for their involvement in cellular signaling, meaning their potential contributions to cancer development are endless [166]. Enrichment of expressed 14-3-3ζ was identified in several cervical cancer cell lines via comparative proteomic analysis with a non-tumorigenic cell line [167]. Furthermore, this same study suggests that 14-3-3ζ causes dysregulation of the cell cycle, driving malignant transformation and determining cell fate. Other models have attempted to explain 14-3-3 function in the context of HPV infection by determining the role of E6 phospho-regulation. In HeLa cells, loss of E6AP increased E6 phosphorylation making it the dominant species in these cells where it is also known that E6 levels drop due to its destabilization. The upregulation of modified E6 is dependent upon transcriptionally active p53 and DNA PK [168]. Although, previous studies showed a connection between the presence of an E6 PBM with a phospho-acceptor site and transcriptional inhibition of p53 by E6. Taken together, these results imply that these proteins are part of a negative feedback loop, where p53 activation prompts phosphorylation of E6, in turn enhancing E6's ability to transcriptionally regulate p53 [163,168]. While the full extent of phospho-E6's roles remain to be established, especially in the context of the other 14-3-3 family members, this is another example of the complex connection erected by E6 and its related proteoforms.

#### 2.3.2 E6∗

Many HPV genes are expressed as polycistronic pre-mRNAs that produce various transcripts through alternative splicing, generating differential patterns in mRNA expression throughout infection [169,170]. Processing of the E6-E7 ORF by alternative splicing is a common feature of hr-HPVs, not found in lr-HPVs [171], resulting in the production of multiple transcripts, containing truncated forms of E6. Of these forms, all hr-HPVs encode the E6∗I, normally referred to as E6∗, splice variant [169]. Originally, it was hypothesized that the sole role of E6∗ was to promote E7 translation. The gap between E6 termination and E7 initiation on the E6/E7 mRNA is not adequate to support efficient translation of E7; however, this is not an issue with the spliced transcripts [102,172,173]. Other studies, however, have shown that E7 is predominantly translated from non-spliced mRNA [174,175]. While existence of the splice variant at the protein level has been a point of contention, there have been several reports suggesting biological activity as a protein [[176], [177], [178]] with detection of protein expression in cervical cancer cell lines [179] and binding assays with synthesized E6∗ peptides (data not yet published). Although, it is worth noting the E6∗ protein has yet to be detected in infected cells *in vivo* [180].

E6∗ shares the first 44 amino acids with full-length E6 and possesses an additional 13 residues gained through the intron removal process [177]. This corresponds to the conservation of only a portion of the E6N domain. Additionally, most proteins contain a hydrophobic (L/M/I)XX(L/I/V)X(L/V/I) motif which is implicated in E6∗’s association with E6-E6AP binding [176]. Other than these features, not much is known regarding the structure or biophysical properties of E6∗. Much like its full-length counterpart, E6∗ exhibits a multitude of functions and, similar to the interplay between other oncoproteins, there is a dynamic relationship between E6 and E6∗ that determines the protein's functional capacities. Investigations have implicated E6∗ independently in the involvement of many activities including p53 and WNT/β-catenin signaling, apoptosis, oxidative stress and DNA damage, degradation of some PDZ substrates, and the inflammatory response [176,177,179,[181], [182], [183], [184]]. In an interesting turn of events, some of these attributes are anti-tumor functions while others have contradictory roles in tumorigenesis. It's been proposed that the function of E6∗, at least in part, is to counteract the oncogenic effects of E6. Depending on the HPV type, the roles of E6∗ differ, a trend observed for all HPV oncoproteins, reflecting the intricate functional networks they weave.

### 2.4 E7—

Upon understanding that the genome of hr-HPVs contain transformative properties [185], the HPV protein E7 was quickly implicated in this process [[186], [187], [188], [189], [190], [191]]. Subsequent studies indicated a complementation between E7 and E6 activity leading to the conclusion that both parties were necessary for transformation [108,192,193]. Current understanding, however, suggests that each oncoprotein possesses independent transformation capabilities that are compounded when combined [194]. Furthermore, each oncoprotein has developed unique tactics to reprogram cells, yet these strategies often converge. Frequently, they involve preying on corresponding cellular processes through different targets, although, the overlap of specific cellular targets also occurs. For example, E6 and E7 both promote cell proliferation by affecting tumor suppressor proteins. E6 facilitates the degradation of p53 via the ubiquitin-proteasome system (UPS), whereas E7 drives cell cycle progression through targeting the tumor suppressor, pRB, either directly, through a shared mechanism of UPS mediated degradation of unphosphorylated pRB [195,196], or through indirect targeting of pocket proteins [197,198]. On the contrary, E7 expression has been shown to stabilize p53 [[199], [200], [201]], possibly through multiple mechanisms [[202], [203], [204]]. Furthermore, studies have demonstrated that E6AP has stabilizing effects on E7 in addition to E6 [205]. The interplay between E6 and E7 has led to the paradigm that the two oncoproteins work in concert to drive the viral life cycle, perhaps even intending to balance one another, as they inadvertently result in carcinogenesis as an unwanted by-product [206].

Similarities between the E6 and E7 oncoproteins extend beyond their shared host targets. E7 is a small protein of 100 amino acids that can be divided and defined by three conserved regions: CR1, CR2, and CR3 [50,207]. This division of primary sequence is analogous to that of adenovirus E1A to which the N-terminus of E7 proteins share sequence and functional homology [188]. These properties are also shared with the SV40 large T antigen and involve a fully conserved LXCXE motif which confers high affinity interaction with pRb [197,[208], [209], [210], [211]]. Whereas in the CR3 region of E7 resides two CXXC motifs partitioned by approximately 30 residues creating a zinc binding domain [[212], [213], [214]], similar to the dual zinc binding domains located at the N- and C-terminals of E6 [113,[215], [216], [217]]. Comparison of E6N, E6C, and E7 zinc binding domain sequences suggests that E7 arose from a duplication event of a singular ancestral E6 domain [218,219]. Said ancestral sequence possibly belonged to a single-domain E6 protein present in the supposed proto-papillomavirus [62], with the discovery of single-domain avian and turtle E6 proteins further supporting this hypothesis [220]. Therefore, it is possible that E6N also stemmed from the duplication and divergence of this precursor [221]. Furthermore, despite low sequence identity between E6N and E6C sequences they share structural homology [222] in contrast to E7 which shares sequence identity with the E6 domain but adopts a significantly different fold [218]. This could indicate that while the proteins stem from a common ancestor, they adopt different functions leading to different protein conformations as they adapt to their niche. A theory reaffirmed by the low sequence conservation of E6 or E7 proteins from various viruses, indicating that the viral type necessitates the form and function of each set of proteins [218]. This is reflected in interactomic studies which have demonstrated that the different types of E6 or E7 proteins possess different interactomes [145,147,149,150,[152], [153], [154],^156^,[223], [224], [225]] and supports the idea that some functions evolved are simply adaptive as opposed to perpetuating viral reproduction [61]. However, the question remains as to whether during this process E6 and E7 have co-evolved, working harmoniously with one another to drive the viral life cycle or, instead, are in competition with one another. Due to the bicistronic organization of the HPV genomes [226] the pair is always present together in infected cells. It stands to reason that this has driven co-evolution of the duo, however, it is possible that the two are not working in alliance and these adaptive functions could include attempts to adapt to selective pressure onset by direct competition between the pair. Overlap in targets could represent attempts to thwart one another and demonstrate functional advantage which instead backfires because the oncoproteins are continuously successful in outcompeting one another. For example, earlier in section 2.4 we described that E7 expression has a stabilizing effect on p53. This could have been a pressure exerted on E6 by E7 that was subverted by E6 through the emergence of the phospho-E6 proteoform and development of transcriptional mechanisms to regulate p53 [168,[227], [228], [229]]. Also mentioned is the stabilizing effect of E6AP on E7. In this study, increases in the level of E6AP were also associated with increases of E7, but these were subject to regulation by E6 [205]. This indicates a potential regulatory loop between the oncoproteins, where E6 can exert control over E7 expression. Taken together, this could represent the remnants of a prior E7 interaction, which was lost after out competition by E6. Therefore as the selective pressure increases, the pair must become more inventive and ultimately the cumulative effects of their ingenuity results in cancer.

## 3 Conclusions

The process of HPV induced carcinogenesis is complex with many players involved. While much progress has been made, aiding our understanding of the respective roles of each oncoprotein, studies often focus on singular features of the singular components: structure, localization, expression levels, and individual targets or pathways. Interactomic studies have begun to open our eyes to the system-wide perturbances caused by these proteins, but much is left to be deciphered regarding how these proteins interact with the host and, potentially more importantly, how they interact with each other. This unholy trinity shares a motive to perpetuate the viral life cycle, and in their efforts to meet this shared objective, it could be as simple as having too many cooks in the kitchen. Their repeated efforts to target mutual cellular mechanisms are too successful, accumulating and leading to the cellular catastrophe that is cancer. Furthermore, it appears that certain host factors possess the power to convey a propensity for carcinogenesis meaning that the trio is not acting alone in determining this fate.

## Funding sources

Work in the Trave lab is funded by institutional support from le Centre National de la Recherche Scientifique (CNRS) and l’Institut National de la Santé et de la Recherche Médicale (INSERM) with projects related to HPV receiving further funding from Ligue contre le Cancer and IMCBio.

## Declaration of competing interest

The authors declare that they have no known competing financial interests or personal relationships that could have appeared to influence the work reported in this paper.

## Data Availability

No data was used for the research described in the article.
